# Editorial: On the Development of Space-Number Relations: Linguistic and Cognitive Determinants, Influences, and Associations

**DOI:** 10.3389/fpsyg.2020.00182

**Published:** 2020-02-14

**Authors:** Krzysztof Cipora, Maciej Haman, Frank Domahs, Hans-Christoph Nuerk

**Affiliations:** ^1^Department of Psychology, University of Tübingen, Tübingen, Germany; ^2^LEAD Research Network, University of Tübingen, Tübingen, Germany; ^3^Faculty of Psychology, University of Warsaw, Warsaw, Poland; ^4^Department of Linguistics, University of Erfurt, Erfurt, Germany

**Keywords:** Spatial-Numerical Association, number processing, spatial biases, cognitive development, Spatial-Numerical Association taxonomy

## Opening Remarks

Tight, bidirectional links between mathematical cognition and spatial processing are documented (Cipora et al., 2015). For instance, there is a relationship between mathematical and spatial development (Young et al.). Individuals who perform well on spatial tasks perform well on math tasks as well (Mix et al., 2016, for a review). Individuals with impairments in mathematical processing are more prone to interference on spatial tasks, even when such tasks do not contain numerical components (Eidlin-Levy and Rubinsten). Apart from correlations between spatial and numerical skills and deficits, there is a broad range of phenomena linking numerical and spatial processing referred to as “Spatial-Numerical Associations” (SNAs). SNAs are not just correlates of numerical processing and math skills, it is also supposed that they may be the key to hidden, deep properties of numerical representations and processes operating on them.

In this context, the question not only of how SNAs correlate with numerical development but also what their role in this development is arises. Lifetime development of SNAs and their functional role is therefore a focus of this Research Topic. As one can see from this collection of 27 papers, there is a considerable variety of SNAs. Our guidance through this variety is based on a taxonomy of SNAs we have proposed and extended (Patro et al., 2014; Cipora et al., 2015, 2018b).

The major distinction in this taxonomy of SNAs is between *Extension and Direction SNAs*. In extension SNAs, numbers are associated with (one- or multi-dimensional) extensions in space: larger numbers are associated with larger extensions. There are two subcategories within extension SNAs. In *Approximate Extension SNAs* “more” in one domain corresponds to “more” in the other one (e.g., in the numerical Stroop task larger font size is associated with larger numerical magnitude, but there is no relationship between specific font size and specific numerical magnitude). In *Exact Extension SNAs*, the accuracy of a relationship between number magnitude and spatial extension is examined. The variable of interest is usually the deviation from the exact isomorphy of numerical and spatial magnitude. For instance, in the number line estimation task the deviation of a marked spatial position on the number line and the position corresponding to the exact numerical magnitude given is analyzed.

In case of *Direction SNAs*, relationships between numbers and specific directions in space (left-right, up-down, near-far) are investigated. The association can either be explicit (as revealed in overt controllable actions such as ordering objects in a certain dimension), or implicit (e.g., a reaction time pattern; Dehaene et al., 1993; Nuerk et al., 2005b). Importantly, several aspects of a number can be associated with space: cardinality, ordinality, functions, and place-value structures.

The rationale for such a taxonomy and distinction is conceptual and is not only aimed at emphasizing peculiarities of different tasks used to measure SNAs. SNA types differ in several fundamental aspects: (1) their relationship with math skill, (2) their potential for being used in interventions, and (3) the extent to which they are prone to situated influences (Cipora et al., 2018c). We are glad to see that papers published in the Research Topic cover all SNA types from the taxonomy (except two types, which we theoretically postulated, but could not find any existing study supporting their existence).

## Direction Implicit SNAs

### Cardinality

This SNA category is the most investigated in the literature and also very well-covered in the current Research Topic. Among other phenomena it considers the SNARC effect (Spatial-Numerical Association of Response Codes; Dehaene et al., 1993; Wood et al., 2008), the hallmark effect showing left-to-right mapping of numerical magnitude representations in Western cultures. Papers published in this volume focusing on this SNA category were either aimed at investigating the foundations of this SNA type in general, or searched for a functional role at different stages of development. In the first group of papers, McCrink and de Hevia outline a new theory on origins of directional SNAs. Their work attempts to integrate opposing views on directionality in SNAs: the nativist (e.g., Rugani et al., 2015; Di Giorgio et al., 2019) and culture related (Shaki et al., 2009; Patro et al., 2016a,b; Patro and Nuerk, 2017). According to their new proposal, left-to-right SNAs are inborn, but in humans they weaken at toddlerhood and then are further (re)shaped by cultural factors. Sosson et al. provide empirical evidence for the developmental trajectory of directional spatial biases in an infrequently used task: random number generation. Adult leftward head movements are related to generating smaller random numbers compared to numbers generated during rightward movements, however, such an effect was not observed in children. SNAs are not only influenced by development *per se*, but also by developmental disorders. Georges et al. looked at the SNARC effect in individuals with ADHD. They found that weaker inhibition capacities were related to a stronger SNARC effect as measured with magnitude-irrelevant parity judgment, and a weaker SNARC effect as measured with a magnitude-relevant magnitude classification task.

Whether a relation between directional SNAs and arithmetic skill exists, is controversial (Cipora et al., 2015, 2018b,c, 2019). Aulet and Lourenco report null, or in one task even negative, correlations between SNA strength and arithmetic skills, corroborating earlier findings that directional SNAs are at least not consistently linked to math skills. However, this does not imply that directional SNAs cannot be used to enhance mathematical understanding as a symbolic tool. Indeed, Thevenot et al. showed that SNAs can enhance memorization of digits in a memory game. Such findings have potential future educational applications.

Another group of papers were not focused on development or associations with math skills, but on the foundations and measurement of SNAs. In a theoretical contribution, Mende et al. discuss the influence of motoric responses. They suggest that the spatial associations of negative numbers might be not a direct measure of their representation, but rather influenced by response paradigm, namely, if an individual has two horizontally aligned response keys or just one. The influence of situated and embodied factors on SNAs seems to be important (Cipora et al., 2018a). Using a 3D virtual setup, Lohmann et al. demonstrate that the strength of the SNARC effect is modulated by perceived physical proximity between numbers and (virtual) responding hands. Finally, language attributes can also influence directional SNAs. Lachmair et al. contrast the role of magnitude and multitude (singular or plural grammatical number) on vertical SNAs, showing that magnitude is a more robust factor.

In sum, papers in this Research Topic (together with other literature) suggest that directional SNAs are multi-facetted in their development, that their relation to arithmetic skills remains ambiguous and that other domain-general factors like motoric or linguistic factors are relevant in their investigation.

### Ordinality

Numerous studies have demonstrated this SNA type, including SNARC-like effects for non-numerical sequences such as days of the week (Gevers et al., 2003) or object position in working memory (van Dijck and Fias, 2011). Dural et al. used a slightly modified paradigm and show a SNARC-like effect for object size in a new memory retrieval paradigm, thereby extending generality of this SNA type across different paradigms.

### Functions

In past years we witnessed a vivid discussion about spatial biases in mental arithmetic: One major finding of debate was the Operational Momentum (OM) Effect (Pinhas and Fischer, 2008; Knops et al., 2009). Pinheiro-Chagas et al. investigated its developmental trajectory in a group of children ages 8–12. They observed a monotonic increase in the size of their OM with age and attributed this finding to increasing reliance on the Mental Number Line representation while performing mental calculations and involvement of attentional processes. Fischer et al. comment on this paper emphasizes multifaceted origins of the OM Effect, which cannot only be accounted for solely by attentional processes. Instead, they argue that their AHAB (arithmetic heuristics and biases) model better accounts for OM development (including the reversed OM in 6-year-olds). The authors of the original paper (Didino et al.) refute the criticism of Fischer et al. by (1) providing arguments that the alternative mechanism of logarithmic compression of the mental number line, on which additions and subtractions are performed was not a strawman argument; (2) stating that heuristics and the attentional account are hardly distinguishable empirically; (3) providing alternative scenarios for which kind of vertical movement should be related to addition and subtraction. At the same time, they call for more precise definitions of the OM and estimation biases induced by the operation sign.

### Place Value

In their taxonomy of interactions between languages and place-value processing, Bahnmueller et al. (esp. Table 1) describe a variety of implicit and explicit place-value effects at three place-value levels: place identification, place-value activation, and place-value computation. Place-value processing can be influenced at all of these levels by various linguistic attributes.

## Direction Explicit SNAs

### Cardinality and Functions

In our taxonomy we postulate the existence of these subcategories, but so far we have not identified any studies investigating them.

### Ordinality

Explicit directional SNAs have been mostly investigated in terms of cultural differences in counting. Based on a large-scale cross-cultural study on five different cultures Bender et al. suggest that the mere focus on main effects of cultural attributes falls short, because characteristics of task and paradigm as well as individual differences need to be considered to obtain a more comprehensive picture.

### Place-Value

In our taxonomy, we have defined place-value processing as a directional SNA because the correct processing of the spatial direction of digits is necessary to assess place-value magnitude (29 vs. 92). Linguistic factors are known to influence multi-digit number processing (e.g., Nuerk et al., 2005a; Moeller et al., 2015; Bahnmueller et al.). Dowker and Li compare English and Hong Kong (L1 Cantonese) children in various tasks. Although the Cantonese-speaking children generally outperformed the English ones, Dowker and Li did not always find specific language effects that could be attributed to the greater transparency of the Chinese counting system and suggest that besides linguistic differences, cultural differences also deserve thorough consideration. Heubner et al. show that congruency effects observed in multi-digit number processing cannot only be reduced to magnitude-related influences. They also show that psychologically parity is not just an exact categorical attribute as it is mathematically. Multiple numerical and non-numerical factors influence parity judgments of two-digit numbers, and some of these influences are language specific as well.

## Extensions—Exact SNAs

This SNA category was also thoroughly investigated in the past and is very well-covered in the Research Topic. Studies on this SNA type fall into three groups. Firstly, some studies have aimed to investigate the nature of this SNA type by proposing new experimental paradigms and developing new theories. Thompson et al. show that finding a specific page in a book correlates with number line estimation scores (even after controlling other variables), and this new task is postulated to tap into the same processes as the traditional number line estimation task (Siegler, 2009). Van't Noordende et al. show that children dynamically change strategies employed when solving the number line task. Secondly, Dowker and Li also utilized the number line estimation task, and show that although Chinese children outperform their English peers in more language-loaded task, the performance on the number line estimation task does not differ between the groups, suggesting less reliance of this SNA on language (see Helmreich et al., 2011 for opposite effects). Thirdly, Sella et al. as well as Opfer et al. focus on the functional role of this SNA type. Sella et al. show that preschoolers' ability to compare Arabic numbers (but not number words) relates to both ordinal and cardinal knowledge as well as the ability to order numbers in space. In the case of number words, magnitude comparison depended only on ordinal knowledge. Opfer et al. show that accuracy of linear mappings of numbers in the number line estimation task relates to multiple measures of memory for numbers and thus may provide a cornerstone or helpful tool for number memory.

All of these dissociations and impacts observed within this SNA type call for more fine-grained analyses and careful generalizations of conclusions.

## Extensions—Approximate SNAs

Papers referring to this SNA category in the present Research Topic were mostly investigating the nature and structure of this SNA type, but also employed novel paradigms. Kucian et al. argue for the presence of a general magnitude system responsible for both spatial and numerical processing and attempt to observe its developmental trajectory. Their empirical evidence suggests that the structure of such a magnitude system is rather complex and consists of dissociated but related representations of continuous and discrete magnitudes. Rugani et al. provide evidence that numerical primes not only influence reaction time patterns but also affect motor execution, especially the reaching component of the movement. In sum, evidence coming from new task types supports SNA construct and criterion validity.

## Not in Taxonomy

Many, but not all the papers in this volume fit into the taxonomy. Nevertheless, they provide important insights into our understanding of human number processing. Pixner et al. investigate the development of the understanding of the cardinality of small numbers and zero. Zero is a special number and its role is hard to overestimate (see Nuerk et al., 2004; Nieder, 2016). Thus, it is crucial to understand the developmental trajectories of the understanding of the zero concept. Eventually, in the opinion piece, Fischer et al. provides multiple arguments for why numbers are embodied concepts. While it plays a crucial role in some extension-exact SNAs (especially embodied number line trainings,Dackermann et al., 2017, for a review), its role for some directional SNAs, and especially place value-processing might be more limited.

## Where Do We Stand: Conclusions and Limitations

Where are we with the SNA research? What are SNAs and what is their function in math skills and number processing? Summarizing existing studies, especially considering papers published within this Research Topic, we propose the following agenda for future SNA research.

Firstly, at least for now, we need to suspend our hopes about making conclusions as to what SNAs are in general since they seem to be a too broad range of phenomena and underlying representations. One can characterize some common features of some but not all SNA types. These common features may partially overlap. However, for the moment, the conclusions about SNAs, and their role need to be limited to specific SNA instances.

Secondly, both previous research (see Cipora et al., 2018a) and papers published in this Research Topic (e.g., Lohmann et al.; Bender et al.) show that SNAs are not stable or fixed, but they can be influenced by factors such as language, culture or even simple experimental manipulation. These inter- and intra-individual differences need to be taken into account when the functional role of SNAs is investigated.

We wish to summarize with the claim that space is an extremely important tool for many aspects of number processing and representation. However, not all SNAs (e.g., Directional Implicit Cardinality SNAs) might be equally important for math skills. We still need to learn when space is important for math, but also when math is important for space: after all, in most of the studies we are looking at correlations. The example in which math skills influence strategies and also performance in spatial tasks has already been demonstrated (see Van ‘t Noordende et al.). For future investigation of the reciprocal interaction between SNA and math skills, it is crucial to specify both the SNAs and math skill under scrutiny, and carefully generalize the conclusions to other SNA types.

## Author Contributions

KC and H-CN wrote the first draft, which was then read, commented, and approved by FD and MH. All authors approved the final version of the manuscript.

### Conflict of Interest

The authors declare that the research was conducted in the absence of any commercial or financial relationships that could be construed as a potential conflict of interest.
